# A Genetically Modified Protein-Based Hydrogel for 3D Culture of AD293 Cells

**DOI:** 10.1371/journal.pone.0107949

**Published:** 2014-09-18

**Authors:** Xiao Du, Jingyu Wang, Wentao Diao, Ling Wang, Jiafu Long, Hao Zhou

**Affiliations:** 1 State Key Laboratory of Medicinal Chemical Biology, Nankai University, Tianjin, China; 2 College of Life Sciences, Nankai University, Tianjin, China; 3 College of Pharmacy, Nankai University, Tianjin, China; Brandeis University, United States of America

## Abstract

Hydrogels have strong application prospects for drug delivery, tissue engineering and cell therapy because of their excellent biocompatibility and abundant availability as scaffolds for drugs and cells. In this study, we created hybrid hydrogels based on a genetically modified tax interactive protein-1 (TIP1) by introducing two or four cysteine residues in the primary structure of TIP1. The introduced cysteine residues were crosslinked with a four-armed poly (ethylene glycol) having their arm ends capped with maleimide residues (4-armed-PEG-Mal) to form hydrogels. In one form of the genetically modification, we incorporated a peptide sequence ‘GRGDSP’ to introduce bioactivity to the protein, and the resultant hydrogel could provide an excellent environment for a three dimensional cell culture of AD293 cells. The AD293 cells continued to divide and displayed a polyhedron or spindle-shape during the 3-day culture period. Besides, AD293 cells could be easily separated from the cell-gel constructs for future large-scale culture after being cultured for 3 days and treating hydrogel with trypsinase. This work significantly expands the toolbox of recombinant proteins for hydrogel formation, and we believe that our hydrogel will be of considerable interest to those working in cell therapy and controlled drug delivery.

## Introduction

By mimicking the biochemical and mechanical properties of native tissue, hydrogels possess hydrated networks of bioactive components [1], such as drugs, proteins, sugars and even cells, leading to contributions to the fields of controllable drug delivery [2], [3], tissue engineering [4], [5], cell culture [6], [7], and others. Within our bodies, cells are known to receive and respond to signals from the crosstalk of extrinsic complexes by proteins, one of the essential building blocks [8]. Thus, protein-based hydrogels can not only provide excellent environments for the cells, but also respond to external stimuli. More and more researchers have focused on the promising applications of protein-based hydrogels, especially in the analytic detection [9], [10] and three-dimensional cell culture [11], [12]. In comparison with traditional 2D monolayer culture, 3D cell culture clearly approaches the natural physiological conditions of the cell. Besides, cell behaviors including migration, morphology and differentiation can be tailored by the exquisite design of the proteins according to the primary amino acid sequence, which makes protein-based biomaterials stand out among this class of materials [13]. For example, cells exhibit expansion and adhesion in gels by integrating adhesive peptide modules such as RGDS and REDV [14].

Moreover, proteins usually serve as cross-linkers via covalent (Michael addition [15], [16], enzyme reaction [17] or site selective conjugation [18], [19]) or non-covalent interactions (specific protein-peptide [20], protein-protein [21], [22] or protein-polysaccharide interactions [23]) in protein-based hydrogels, which require them to have multiple binding sites to their ligands. Some specific amino acid side chain groups such as the lysine’s ε-amine and cysteine’s sulphydryl endow favourable targets for cross-linking reactions [24], [25].

Recently, Yang’s group reported a novel biohybrid hydrogel containing a polymer of 4-armed-PEG-Mal and tetrameric recombinant protein (ULD) for 3D cell culture of NIH 3T3 [11]. In their gel, the cysteines required for hydrogel formation was inherent and exactly coincided at the outer surface of the protein. However, there are no endogenous cysteines available for bioconjugation to the polymers or peptides of many proteins, or the cysteines hide in the inner part of the proteins. Such cases are surely limiting the range of proteins that can be chosen for hydrogel formation and subsequent applications. In order to overcome that problem,we rationally replaced two or four amino acids with cysteine in TIP1 with no inherent cysteines [26] according to its crystal structure and used rheology and SEM to characterise the corresponding hydrogels that were formed by thiol-maleimide reaction. We also incorporated a bioactive peptide, GRGDSP, at the C-terminus of TIP1 2C and uncovered its roles in cell spreading and proliferation in the gel by Live-dead assay and CCK-8 assay.

## Materials and Methods

### Materials

All molecular cloning reagents were obtained from TIANGEN (Beijing, China). The chemical reagents used for protein purification were obtained from VETEC (Sigma, USA). Maleimide-end-capped four-armed poly (ethylene glycol) was purchased from Laysan Bio (Arab, AL). AD293 (Adeno-X 293) cells were purchased from Clontech (Takara, Japan). Dulbecco’s Modified Eagle Medium and fetal bovine serum (FBS) were purchased from GIBCO (Life Technologies, USA) and Hyclone (Thermal Scientific, USA), respectively. Trypsinase (0.25%) + EDTA and penicillin/streptomycin were purchased from Invitrogen (Life Technologies, USA). The Live/Dead Viability Kit was purchased from Invitrogen (Life Technologies, USA). The Cell Counting Kit-8 was obtained from Beyotime (Jiangsu, China). All other commercial chemicals were of reagent grade or better. Ultrapure water was used for all experiments.

### Protein expression and purification

The mutated DNA fragment (TIP1 4C, TIP1 2C or TIP1 2C RGD) was first amplified by polymerase chain reaction (PCR) by using the DNA fragment of wild type human TIP1 as the template along with the corresponding mutated primers (Table S1), then the PCR products were ligated into an in-house modification of the pET32a (Novagen) vector. After transformation and screening for the positive clones, the plasmids were extracted for the next protein expression. The Trx-His_6_-tag was contained in the resulting proteins’ N-termini.

BL21(DE3) *Escherichia coli* cells were cultured in LB medium (10 g/L NaCl, 10 g/L tryptone, 5 g/L yeast extract) at 37°C until the OD_600_ reached 0.6, and then a final concentration of 0.3 mM isopropyl-β-D-thiogalactoside was added to induce protein expression at 16°C for approximately 16 hours. After being centrifuged at 5,000 rpm for 15 minutes, the supernatant was removed and the *Escherichia coli* cells were resuspended in binding buffer (50 mM Tris-HCl, pH 8.0, 200 mM NaCl and 5 mM imidazole). The cells were then lysed by AH-1500 (ATS Engineering Limited) and the lysates were centrifuged at 18,000 rpm for 30 minutes. The supernatant was loaded onto a Ni-NTA-Agarose column that was equilibrated with binding buffer. The Trx-his_6_-tagged proteins were eluted with elution buffer (50 mM Tris-HCl, pH 8.0, 200 mM NaCl and 500 mM imidazole). The eluted proteins were then purified by HiLoad 26/60 Superdex 200 column (GE Healthcare) in T_50_N_200_ buffer (50 mM Tris-HCl, pH 8.0, and 200 mM NaCl). After identifying the protein peak by SDS-PAGE gel, the Trx-his_6_-tagged target protein was collected and then the N-terminal Trx-His_6_-tag was cleaved overnight with PreScission Protease. The digested protein was passed through a HisTrap HP column (GE Healthcare) to remove the protease and tag. Finally, the target protein was loaded onto a HiLoad 26/60 Superdex 200 size exclusion column and eluted with PBS buffer (NaCl 137 mmol/L, KCl 2.7 mmol/L, Na_2_HPO_4_ 4.3 mmol/L, and KH_2_PO_4_ 1.4 mmol/L, pH 7.4). A Superose 12 10/300 column (GE Healthcare) was used to identify the conformation of the final proteins with different concentrations. By following the above procedure, we acquired the TIP1 4C (T10C, S42C, S101C and S113C), TIP1 2C (S101C and S113C) and TIP1 2C RGD proteins (by incorporating the bioactive peptide GRGDSP into the C-terminus of TIP1 2C).

### Hydrogel formation

80 mg/ml of 4-armed-PEG-Mal in PBS buffer and 40 mg/ml of protein TIP1 4C (TIP1 2C or TIP1 2C RGD) in PBS buffer were prepared as stock solutions. For the TIP1 4C gel, 100 µL of PBS buffer and 100 µL of 4-armed-PEG-Mal stock solution were added to 200 µL of TIP1 4C stock solution. A gel of the desired concentration was formed immediately after being kept at room temperature (both final concentrations of the 4-armed-PEG-Mal and the protein in the gel were 2.0 wt%). Other protein-based hydrogels were also formed by this method.

### Rheology

A rheology test was performed on an AR 2000ex system (TA instrument). A 40 mm parallel plate was used with a 400 µm gap during the experiment. For the dynamic time sweep, 250 µL of PBS buffer, 250 µL of 4-armed-PEG-Mal stock solution and 500 µL of protein stock solution were directly transferred onto the rheometer and then analysed at the frequency of 1 rad/s and the strain of 1%. The dynamic strain sweep was performed in the 0.1%–10% region at a frequency of 1 rad/s and the strain value in the linear range was chosen for the following dynamic frequency sweep. The gel was also characterised by the dynamic frequency sweep in the 0.1–100 rad/s region at the strain of 1%.

### Scanning electron microscopy (SEM)

A thin layer of hydrogel was cast on a silica wafer that was cleaned by sonication in ethanol for 10 minutes and then freeze-dried in a lyophiliser overnight. A layer of gold was spluttered on the sample by vacuum spray to produce a conductive surface. The SEM analysis was conducted on a Hitachi X650 system (Japan) operating at 15 kV.

### 3D Cell culture

AD293 cells were used in this study. The cells were cultured in complete Dulbecco’s Modified Eagle Medium (DMEM) supplemented with 10% fetal bovine serum, 100 units/mL penicillin, and 100 µg/mL streptomycin. Before hydrogel formation, 4-armed-PEG-Mal powder and syringe filters with 0.22 µm apertures were sterilized by UV light on a superclean worktable for 60 minutes, and PBS buffer and protein were then filtered by using the above filters. The cells were separated by trypsinase (0.25%) digestion, followed by centrifugation at 800 rpm for 5 minutes and resuspended in DMEM plus 10% FBS and 1% penicillin/streptomycin. 4-armed-PEG-Mal and cells (the final cell density was 1,000,000 cells/ml hydrogel) in 25 µL of DMEM medium was mixed with an equal volume of protein and then transferred to 96-well plates (50 µL of gel per well). Half an hour later, 100 µL of DMEM medium was added to the top of the hydrogel. The 96-well plate was maintained in a 37°C/5% CO_2_ incubator.

### Live-dead assay

The viability of encapsulated cells was tested with a live-dead assay at designated times. The cell-gel constructs were washed three times with PBS buffer for the purpose of buffer exchange. After removing the PBS buffer for the last time, 100 µL of live/dead solution containing 4 µM EthD-1 (ethidium homodimer-1) and 2 µM calcein AM was added onto each cell-gel construct. After 30 minutes of incubation at 37°C with 5% CO_2_, the staining solution was removed. The constructs were observed by using a Nikon Eclipse TE2000-U inverted fluorescence microscope with excitation filters of 450–490 nm (green, calcein AM) and 510–560 nm (red, EthD-1). The confocal analysis was performed on a Leica TSC SP8 system (Germany).

### CCK-8 assay

**A** CCK-8 assay was performed to quantify cell proliferation in the cell-gel constructs at designated time points. After a 3D culture had been established using the above standard procedure, each cell-gel construct was washed with complete cell culture medium three times and mixed with 100 µL of 10% CCK-8 agent (v/v) in serum-free DMEM. The plates were incubated in a 37°C/5% CO_2_ incubator for 4 hours. The absorbance at 450 nm was measured by microplate reader (MultiskaniMark, Bio-Rad, USA). The experiment was performed five times and the SD was then determined.

### Cell Recovery

To identify the growth state of AD293 cells that were separated from TIP1 2C RGD gel, we separated the cells from the cell-gel construct by trypsinase digestion. Trypsinase was added onto the gel after 3 days of 3D culture. After incubating for 5 minutes at 37°C, the resulting solution was transferred into an Eppendorf tube and centrifuged for 5 minutes at room temperature at 800 rpm. The supernatant was discarded and 200 µL of the complete cell culture medium was used to resuspend the cells. The individual collected cells were counted and reseeded on a conventional 96-well culture plate at a density of 10,000 cells/well. The cells were observed at 2 days post culture by ordinary inverted microscope.

## Results

### Protein purification and hydrogel preparation

**Figure 1:**
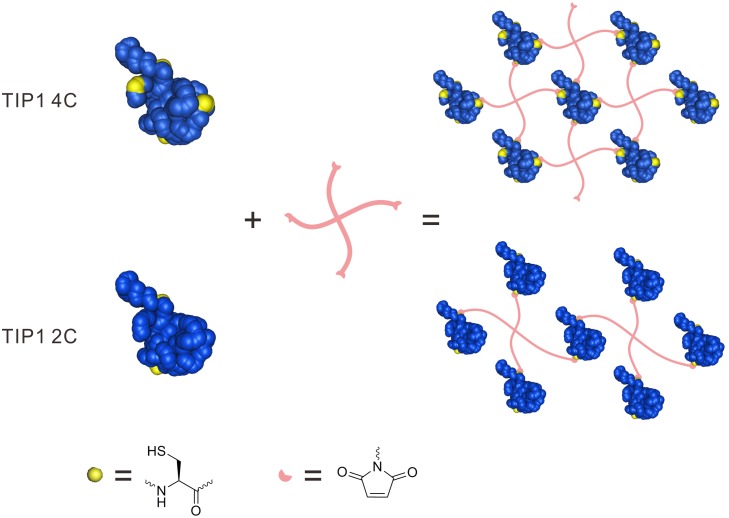
A schematic diagram of hydrogel formation. The Michael addition between maleimides of 4-armed-PEG-Mal and thiols in each mutant protein leads to the formation of 3D networks for hydrogelations. The blue balls represent TIP1 protein. The yellow balls represent amino acids that were replaced by cysteine in TIP1. The pink lines represent 4-armed-PEG-Mal.

**Figure 2:**
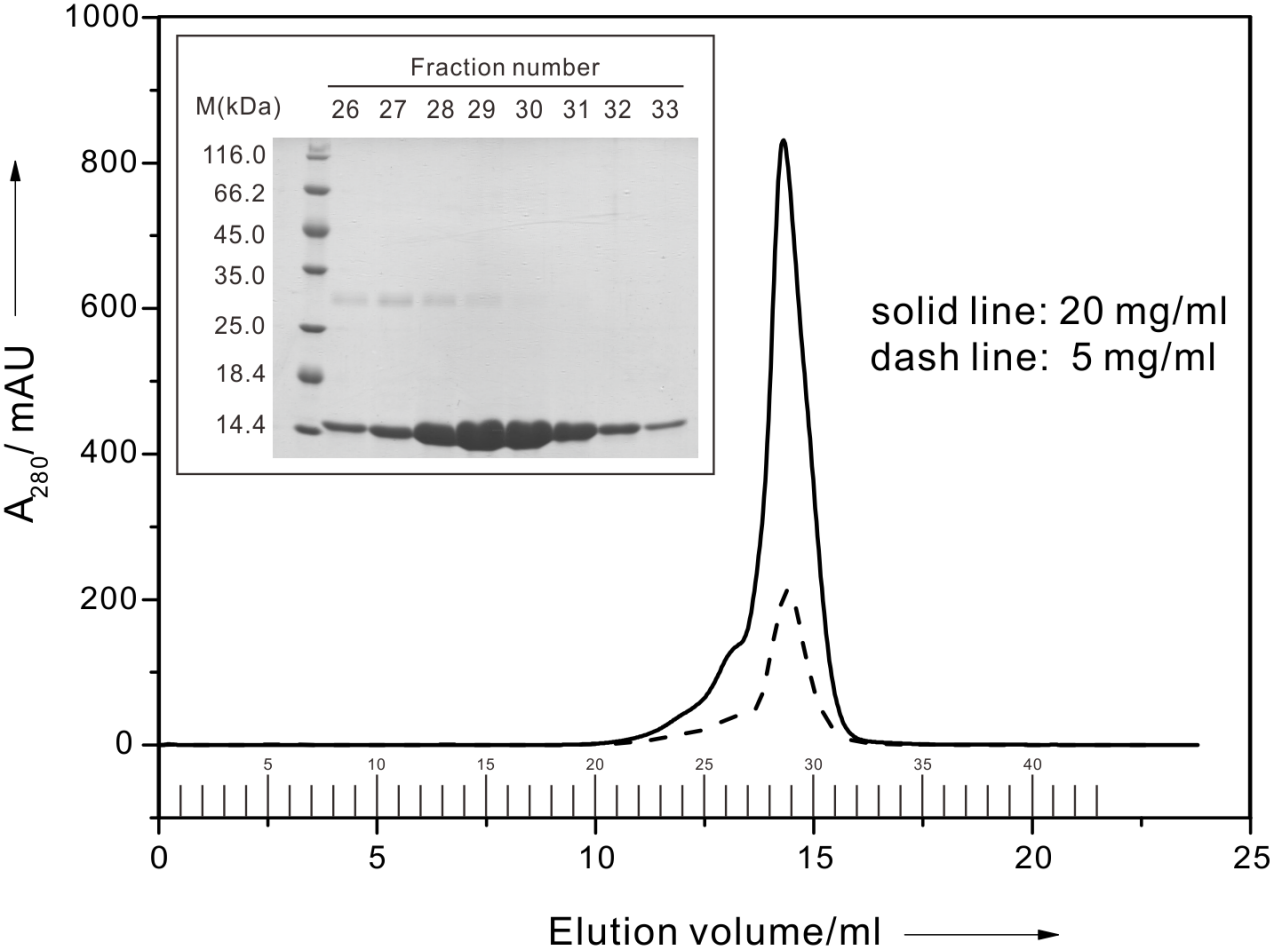
The purification of TIP1 2C. The size-exclusion chromatography of TIP1 2C was performed in a Superose 12 10/300 at two concentrations. Inset: A 20% SDS-PAGE gel result for each fraction.

The protein used for hydrogel formation should be easily purified and have an impressively high yield, such as that of TIP1 [26]. After ensuring that no endogenous cysteine was present and analysing the protein’s crystal structure [26], we genetically modified four amino acids to cysteine at the outer surface (T10C, S42C, S101C and S113C) for further hydrogelation (Figure 1). The TIP1 4C protein yield was comparable to that of TIP1; however, if the purified TIP1 4C (Figure S1) was placed at room temperature for approximately 15 minutes, it was unstable and it precipitated, especially when TIP1 4C was present at a high concentration (approximately 10 mg/ml). We also acquired the TIP1 2C (S101C and S113C) gene by employing the above molecular cloning process. When compared with TIP1 4C, TIP1 2C (Figure 2) had better stability at a high concentration (even at approximately 20 mg/ml) because it had fewer thiols in its monomer. To promote cell spreading, we genetically modified a hydrophilic peptide called GRGDSP to the C-terminus of the TIP1 2C protein and the purification of TIP1 2C RGD was shown in Figure S2.

**Figure 3:**
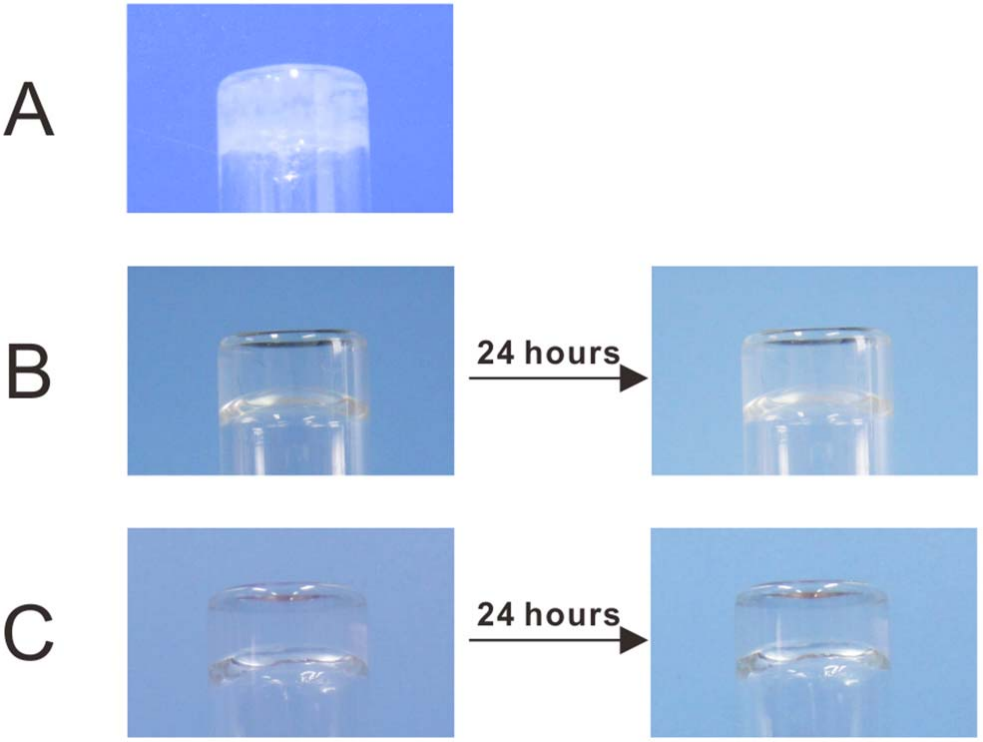
Hydrogel formation and stability. Optical images of three resulting hydrogels formed by 2.0 wt% of 4-armed-PEG-Mal and 2.0 wt% of the corresponding protein. A, TIP1 4C gel; B, TIP1 2C gel; and C, TIP1 2C RGD gel. Both TIP1 2C gel and TIP1 2C RGD gel remained transparent after 24 hours.

The thiols on the outer surface of genetically mutated TIP1 could react with maleimided PEG by Michael addition [16], as indicated by the high molecular weight polymer in the SDS-PAGE gel (Figure S3), thus leading to hydrogel formation. If we adjusted the final concentration of 4-armed-PEG-Mal to 2.0 wt%, the minimum final concentration of TIP1 4C required for hydrogelation was 0.5 wt%. Upon mixing two solutions of 4-armed-PEG-Mal and TIP1 4C (the final concentration of both components was 2.0 wt%), the TIP1 4C gel was formed rapidly and became turbid after 5 minutes (Figure 3A). Such phase separation indicated it was not suitable for future characterisation and 3D cell culture. The mechanism of TIP1 2C gel formation is similar to that of TIP1 4C gel, and the minimum final concentration of TIP1 2C needed for hydrogel formation was 1.0 wt%. TIP1 2C gel (the final selected concentration of both components was 2.0 wt%) could still stay transparent after 24 hours (Figure 3B), which ensured its promising application in 3D cell culture. TIP1 2C RGD gel exhibited the same characteristics on the minimum final concentration for hydrogelation and appearance (Figure 3C). Besides, If we separately mixed PEG and the above proteins pre-treated with small molecule maleimide (the final concentration of both components was 2.0 wt%), none of them formed hydrogel (Figure S4).

### Hydrogel characterization by rheological measurement and scanning electron microscopy

**Figure 4:**
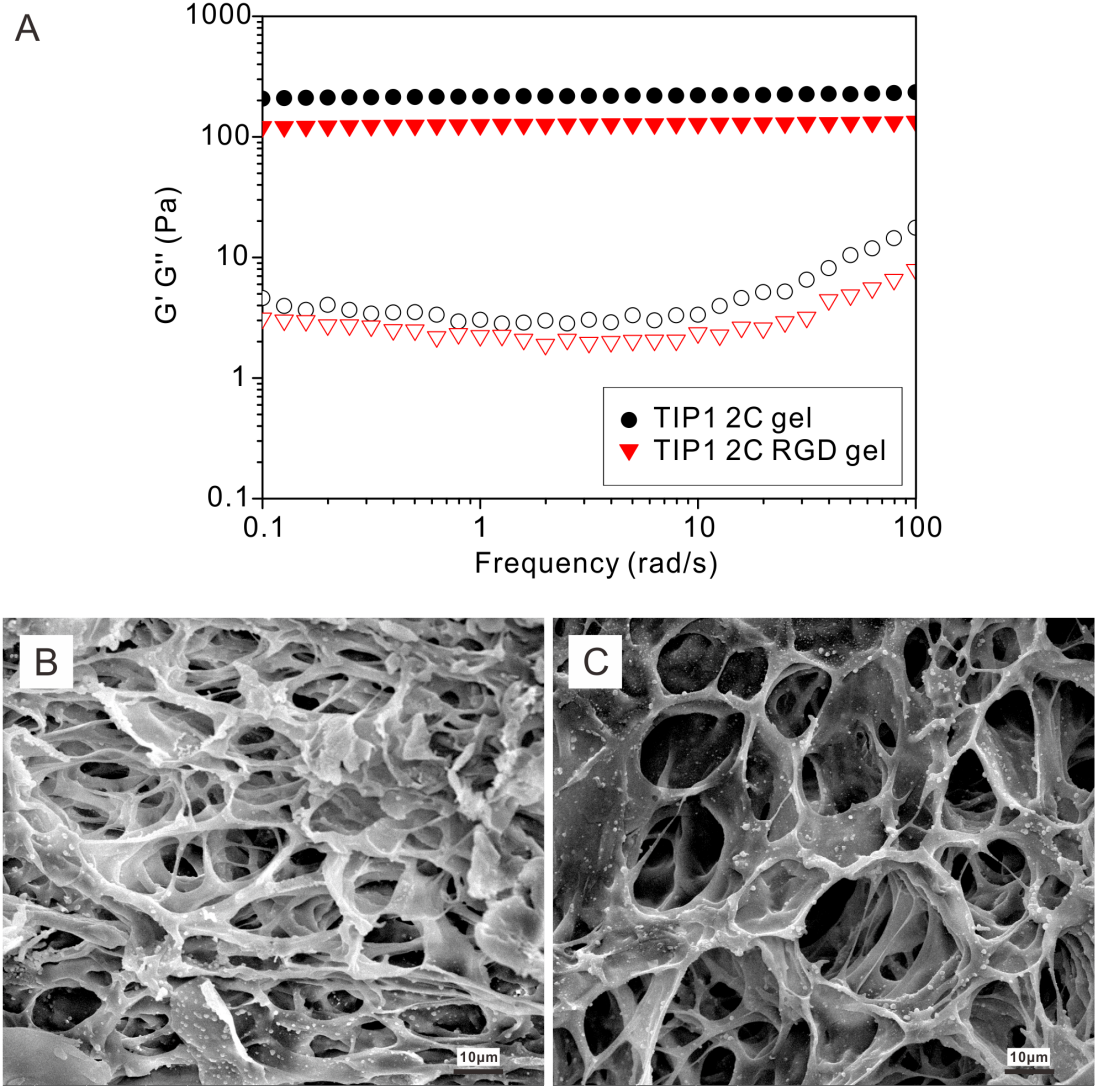
Hydrogel characterization. A, A rheological measurement in dynamic frequency sweep mode at the strain of 1% for each gel containing 2.0 wt% of 4-armed-PEG-Mal and 2.0 wt% of the protein. Closed symbols: elasticity (G’) values and open symbols: viscosity (G”) values. Circles: TIP1 2C gel and triangles: TIP1 2C RGD gel. B, An SEM image of the TIP1 2C gel. C, An SEM image of the TIP1 2C RGD gel.

We used rheological measurements to study the kinetics of TIP1 2C gel and TIP1 2C RGD gel containing 2.0 wt% of 4-armed-PEG-Mal and 2.0 wt% of the corresponding proteins, respectively. A dynamic strain sweep at a frequency of 1 rad/s was first performed to set the strain value for the following sweeps (Figure S5). As shown in Figure S6, a dynamic time sweep was then performed at the strain of 1% and the frequency of 1 rad/s. Two types of gels formed rapidly, as indicated by the elasticity (G’) values dominating the viscosity (G”) values right after mixing the components. The G’ values kept slowly increasing and reached a plateau at 1,200 seconds. The final G’ values of the gels were approximately 210 Pa and 120 Pa for the TIP1 2C and TIP1 2C RGD gels, respectively. Followed by the dynamic time sweep, the dynamic frequency sweep was conducted at the strain of 1% (Figure 4A). The G’ values were almost invariant as the frequency increased, and the G” values were apparently rising from 10–100 rad/s. Moreover, the G’ value in the TIP1 2C gel was more than that of the TIP1 2C RGD gel, which implied a weaker hydrogel for the TIP1 2C RGD gel.

Scanning electron microscopy (SEM) provided useful information about the microstructure of freeze-dried hydrogels [27]. Similar to other reports about protein-based hydrogel, both gels in this study possessed a 3D loose and porous structure (Figure 4B and 4C). The pores were approximately 11.5 µm in the TIP1 2C gel, and they were bigger in the TIP1 2C RGD gel at 16.5 µm. These pores interlaced with one another to form three dimensional networks.

### 3D cell culture and the determination of cell viability and proliferation

**Figure 5:**
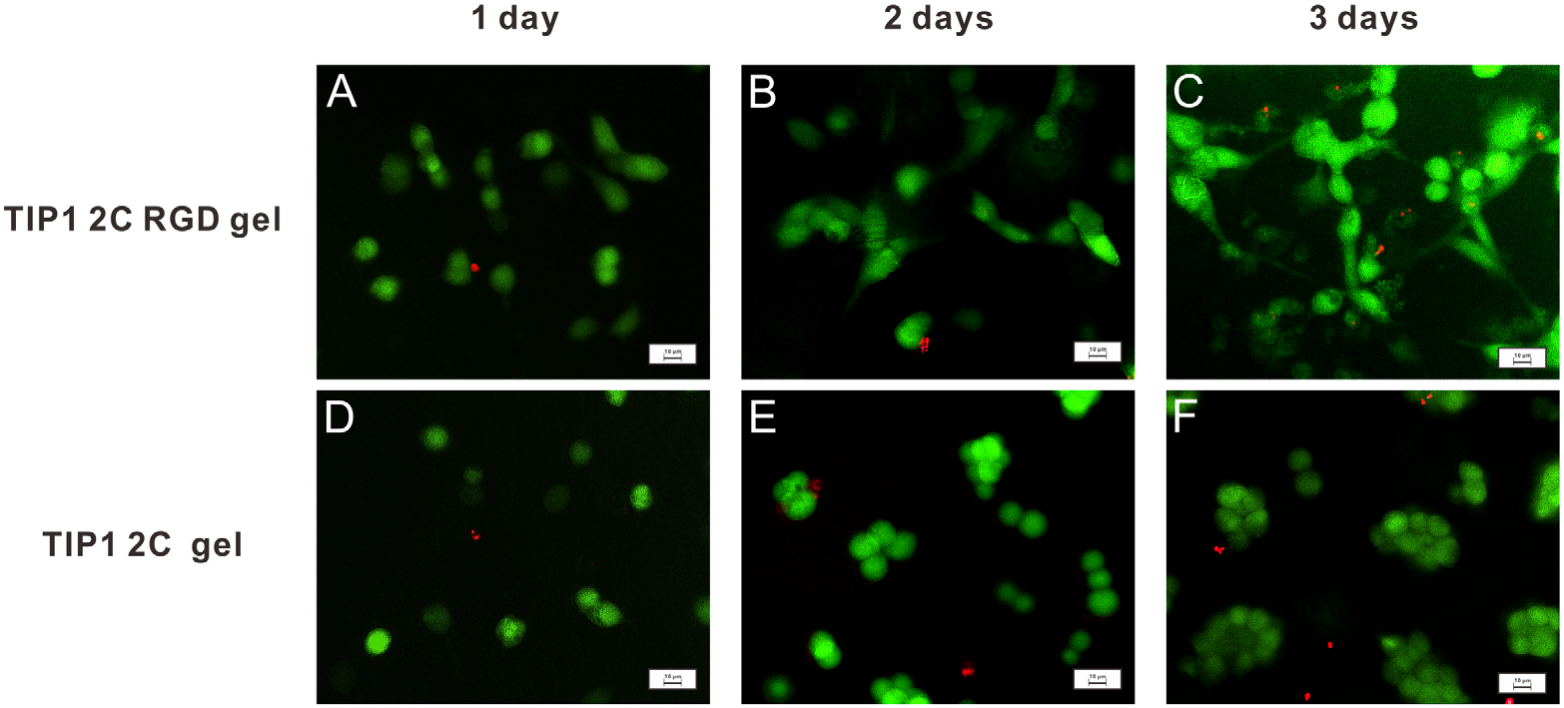
Determining cell viability. A live-dead assay of AD293 cells cultured in TIP1 2C RGD (top) and TIP1 2C (down) hydrogels at different time points. A and D, day 1; B and E, day 2; and C and F, day 3. Live cells were stained green and dead cells were stained red. Magnification: 20×.

We then investigated whether the TIP1 2C gel or the TIP1 2C RGD gel was suitable for 3D cell culture. A DMEM solution containing 1,000,000 cells/ml and 2.0 wt% of 4-armed-PEG-Mal was mixed into PBS solution containing 2.0 wt% of TIP1 2C or TIP1 2C RGD. The mixing-induced hydrogelation process was convenient and rapid, which guaranteed the homogeneous encapsulation of AD293 cells, as demonstrated by the confocal assay (Figure S7). We performed a live-dead assay to determine the cell viability of the gels. Most of the AD293 cells were alive during the 3-day culture period, as indicated by the cells that were stained green, and the dead cells exhibited a red colour (Figure 5). More importantly, cells cultured in the TIP1 2C RGD gel were spreading, polyhedral or spindle-shaped relative to the TIP1 2C gel with cell clusters. The cell densities of both gels kept rising over the 3-day culture period. We quantified the cell proliferation by using a CCK-8 assay. As shown in Figure S8, the number of metabolically active cells persistently increased over the 3-day culture period in TIP1 2C gel. The optical density (OD) in this gel was 1.33, 1.57 and 1.76 at days 1, 2 and 3, respectively. A similar trend was observed in TIP1 2C RGD gel.

TIP1 2C RGD gel could be converted into a clear solution by treating with trypsinase at 37°C for 5 minutes. Therefore, we were able to recovery the AD293 cells after 3 days of culture in gel by trypsinase digestion and centrifugation. The cells that were separated from the cell-gel construct following the above method grew well on normal 96-well culture plates (Figure S9).

## Discussion

The Michael addition is attractive for cell encapsulation because of its mildness and close approximation of physiological conditions [28], [29]. PEG, which has been conjugated to various proteins to form a hydrogel scaffold, is the most widely used polymer in tissue engineering [30], [31]. Among the genetically modified proteins we used, TIP1 4C was unstable, which may have been caused by intermolecular aggregation through nonspecific disulfide bond formation. Although the two mutated serines in TIP1 2C were close to each other in the primary structure, there still existed a certain distance between them in the tertiary structure [26]. Fewer cysteines in the protein oligomer rendered more stability to its corresponding hydrogel, which ensured promising applications such as 3D cell culture. Therefore, rationally designing the mutated sites based on the protein structure is crucial.

A suitable mechanical property is essential to the hydrogel for cell culture. Both TIP1 2C and TIP1 2C RGD gels exhibited frequency dependence and weak mechanical properties, which was a universality of protein-based hydrogels [32]. However, these gels exhibited an apparent difference in 3D cell culture. The noticeable difference between the two gels was related to the modified RGD active peptide. Thus far, peptide ligands that promote cell adhesion [33], [34] or are sensitive to matrix metalloproteinase cleavage [35], [36] have been used extensively to render hydrogels with new features.

Furthermore, when compared with the two gels we used in the CCK-8 assay, the higher OD values in the TIP1 2C RGD gel suggested that incorporating the RGD peptide could partly improve the property of the hydrogel for cell proliferation, adhesion and spreading, which was in accordance with the live-dead assay result. As a result of protein degradation and hydrogel erosion after 3 days of culture, cell recovery should be performed at an appropriate time.

## Conclusions

In summary, we have successfully produced in situ gelating hybrid hydrogels composed of genetically modified recombinant proteins and 4-armed-PEG-Mal. Although the AD293 cells continuously divided over the 3-day culture period in the both TIP1 2C and TIP1 2C RGD gels, they displayed a spreading morphology in the latter one, which demonstrated that the TIP1 2C RGD gel could provide an excellent and biocompatible microenvironment for the 3D cell culture of AD293 cells. The cells that were recovered from the TIP1 2C RGD gel grew well in the conventional 2D cell culture, suggesting its great potential for large-scale culture. We anticipated that this hydrogel would have broad applications in many areas, such as the delivery of cells for cell therapy, controlled drug delivery, and tissue engineering, which was studied in the next step.

## Supporting Information

Figure S1 **The purification of TIP1 4C.** The size-exclusion chromatography of TIP1 4C was performed in a Superose 12 10/300 at two concentrations. Inset: A 20% SDS-PAGE gel result for each fraction.
(TIF)

Figure S2 **The purification of TIP1 2C RGD.** The size-exclusion chromatography of TIP1 2C RGD was performed in a Superose 12 10/300 at two concentrations. Inset: A 20% SDS-PAGE gel result for each fraction.
(TIF)

Figure S3 **Identifying the Michael addition reaction.** A 20% SDS-PAGE gel result for purified proteins and their corresponding hydrogels.
(TIF)

Figure S4 **Hydrogel formation test.** Optical images of three mixtures containing PEG and corresponding proteins pre-treated with small molecule maleimide. A, TIP1 4C; B, TIP1 2C; and C, TIP1 2C RGD.
(TIF)

Figure S5 **A dynamic strain sweep of the gels.** A rheological measurement in dynamic strain sweep mode at the frequency of 1 rad/s for each gel containing 2.0 wt% of 4-armed-PEG-Mal and 2.0 wt% of the protein. Closed symbols: elasticity (G’) values and open symbols: viscosity (G”) values. Circles: TIP1 2C gel and triangles: TIP1 2C RGD gel.
(TIF)

Figure S6 **A dynamic time sweep of the gels.** A rheological measurement in dynamic time sweep mode at the frequency of 1 rad/s and the strain of 1% for each gel containing 2.0 wt% of 4-armed-PEG-Mal and 2.0 wt% of the protein. Closed symbols: elasticity (G’) values and open symbols: viscosity (G”) values. Circles: TIP1 2C gel and triangles: TIP1 2C RGD gel.
(TIF)

Figure S7 **The cell distribution in both gels.** Confocal images of 3D cell cultures in the hydrogels at day 1. Cells were distributed evenly in both gels. A, TIP1 2C gel and B, TIP1 2C RGD gel.
(TIF)

Figure S8 **Determining cell proliferation.** The cell proliferation rate of AD293 was evaluated by CCK-8 assay. Two asterisks (**) indicate a p value smaller than 0.01 (p<0.01). Three asterisks (***) indicate a p value smaller than 0.001 (p<0.001), n = 5.
(TIF)

Figure S9 **Cell Recovery.** A, 200 µL of the TIP1 2C RGD gel was treated with 100 µL of trypsinase (2.5 µg/mL) for 5 minutes, and then 200 µL of PBS was added. B, The solution was then centrifuged at 1000 rpm for 5 minutes and no precipitation was observed. C, The AD293 cells were separated from the cell-gel construct by adding trypsinase and then centrifuging. The cells could grow well on a conventional 96-well culture plate.
(TIF)

Table S1 **The primers used for mutations in this study.** The restriction sites are underlined.
(DOC)
