# Supplementary figures and images for: A Genetically Modified Protein-Based Hydrogel for 3D Culture of AD293 Cells
Source: PLoS One. 2014 Sep 18;9(9):e107949. doi: 10.1371/journal.pone.0107949 (PMC4169439; doi:10.1371/journal.pone.0107949)

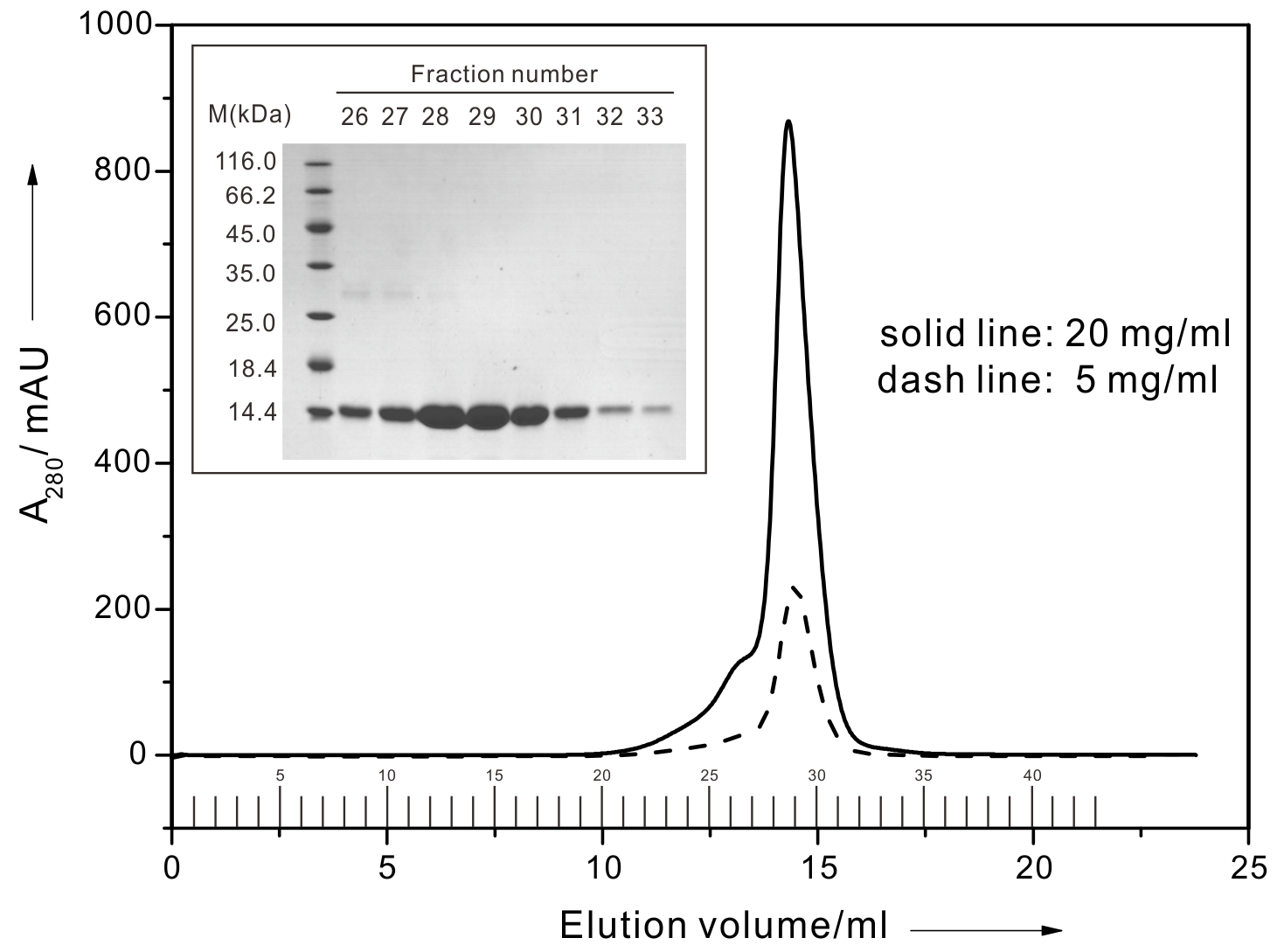

Supplement: Figure S1 — The purification of TIP1 4C. The size-exclusion chromatography of TIP1 4C was performed in a Superose 12 10/300 at two concentrations. Inset: A 20% SDS-PAGE gel result for each fraction. (TIF) [file pone.0107949.s001.tif]

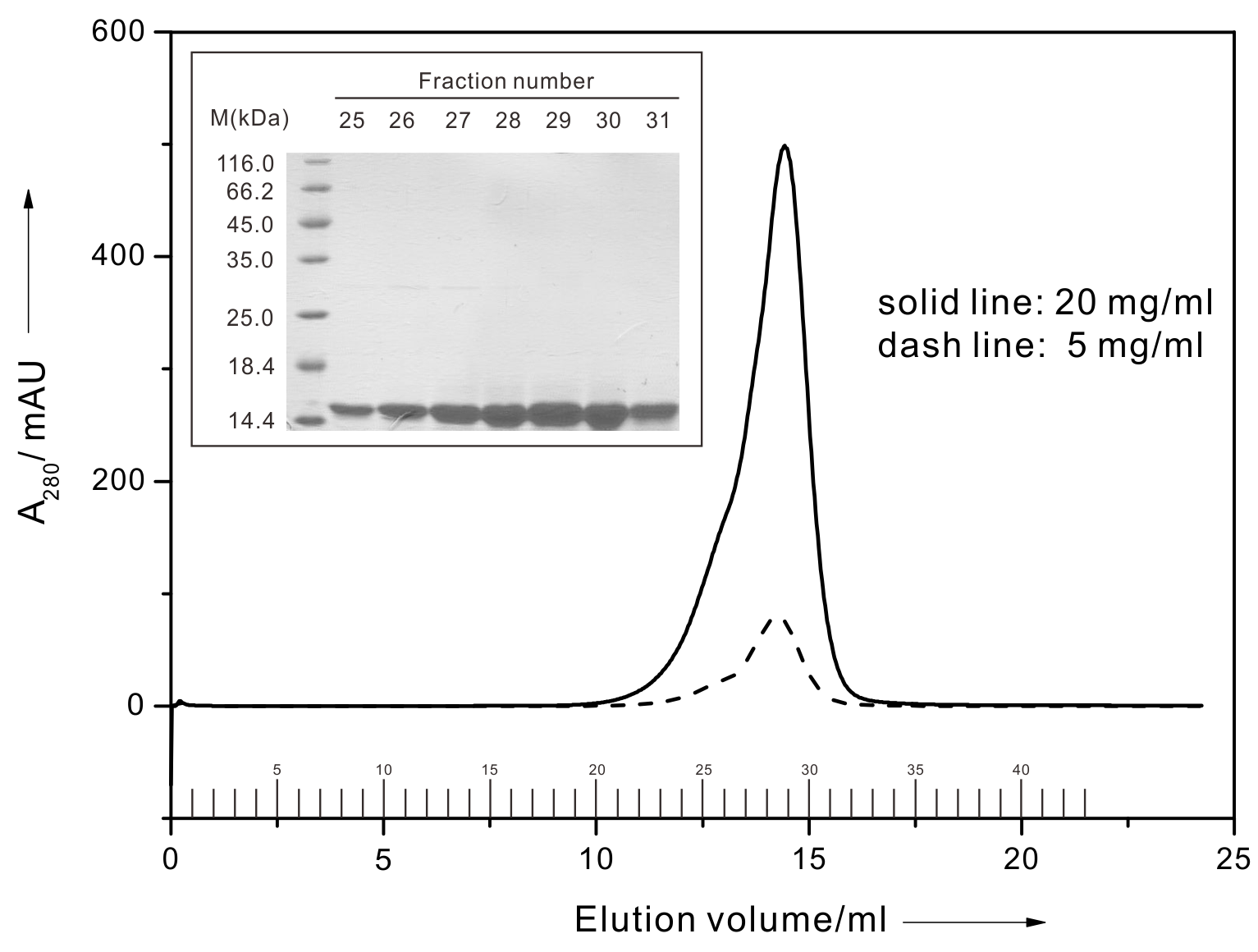

Supplement: Figure S2 — The purification of TIP1 2C RGD. The size-exclusion chromatography of TIP1 2C RGD was performed in a Superose 12 10/300 at two concentrations. Inset: A 20% SDS-PAGE gel result for each fraction. (TIF) [file pone.0107949.s002.tif]

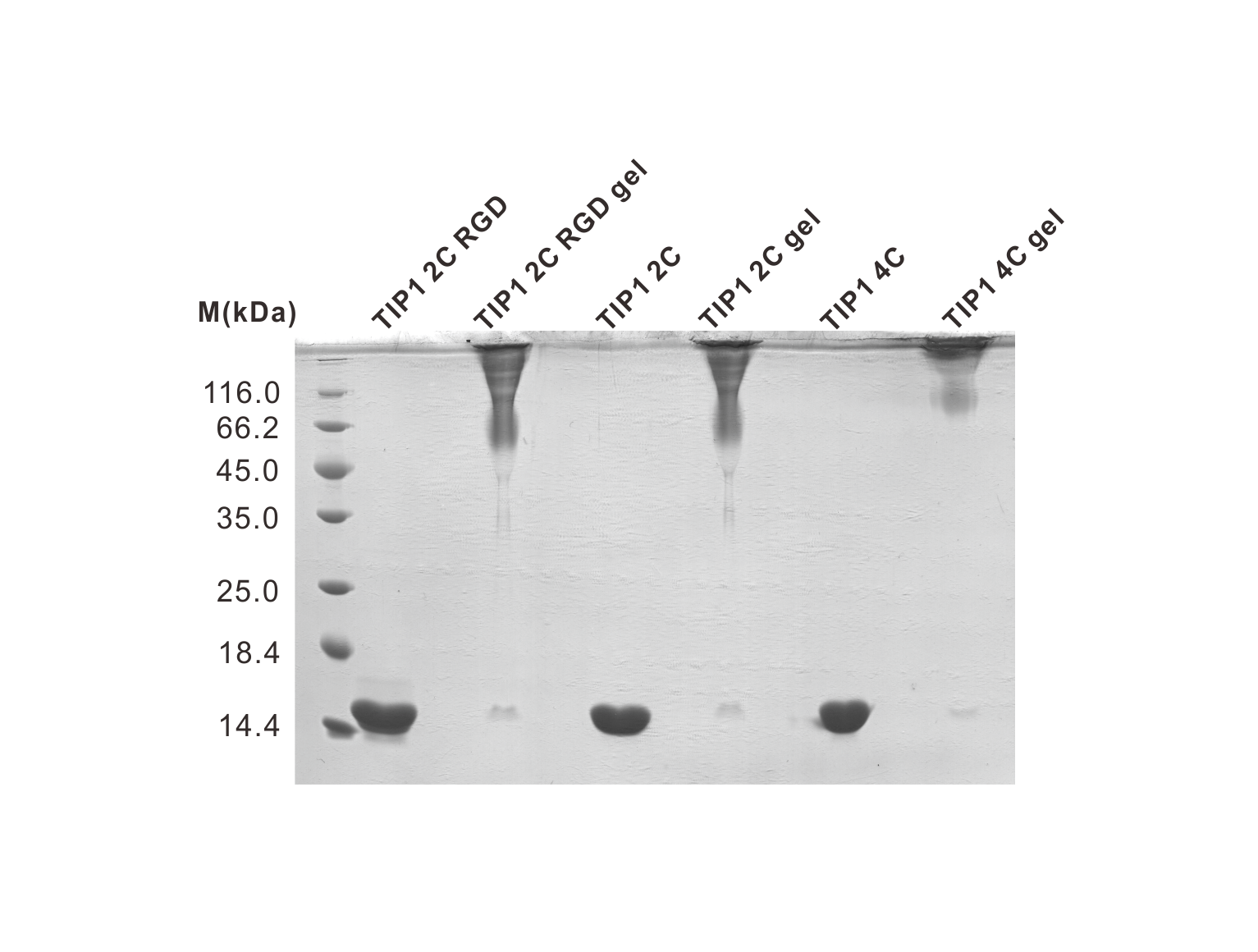

Supplement: Figure S3 — Identifying the Michael addition reaction. A 20% SDS-PAGE gel result for purified proteins and their corresponding hydrogels. (TIF) [file pone.0107949.s003.tif]

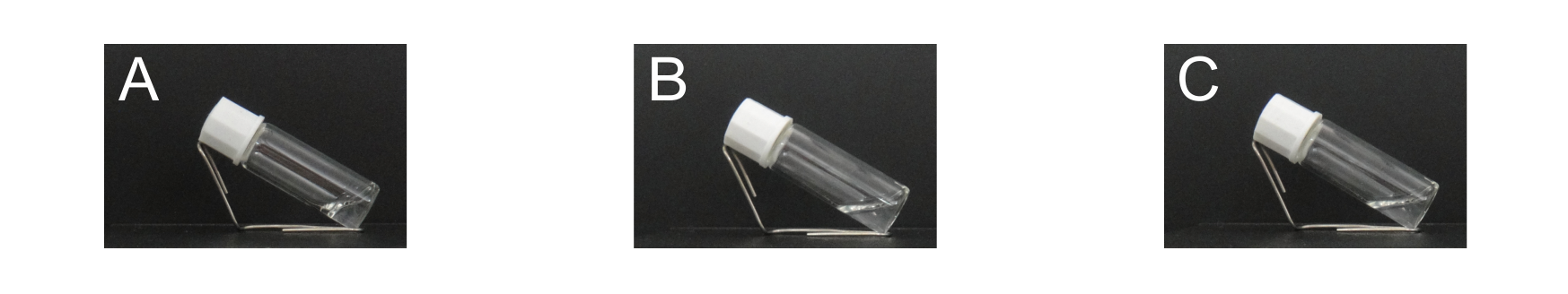

Supplement: Figure S4 — Hydrogel formation test. Optical images of three mixtures containing PEG and corresponding proteins pre-treated with small molecule maleimide. A, TIP1 4C; B, TIP1 2C; and C, TIP1 2C RGD. (TIF) [file pone.0107949.s004.tif]

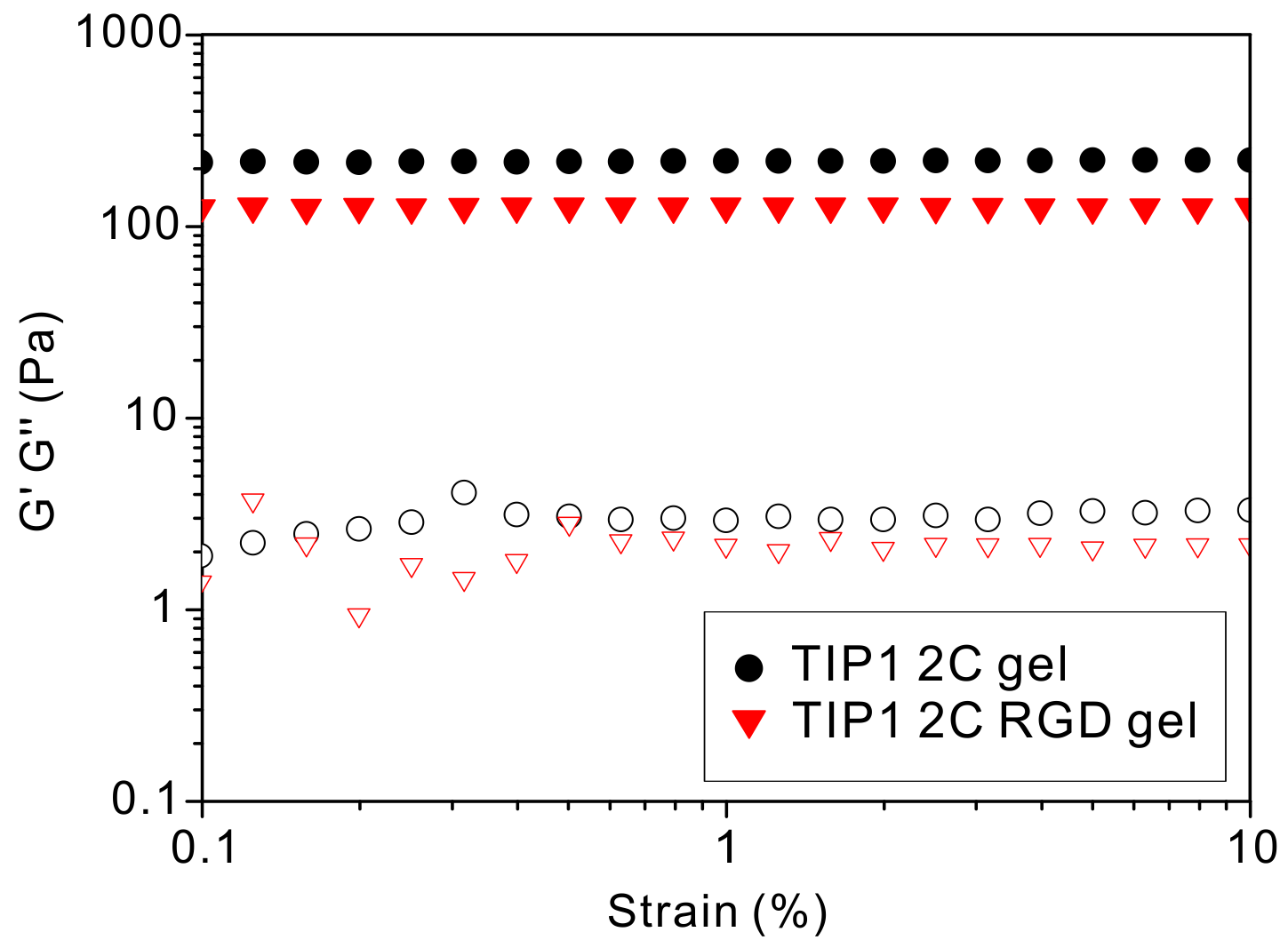

Supplement: Figure S5 — A dynamic strain sweep of the gels. A rheological measurement in dynamic strain sweep mode at the frequency of 1 rad/s for each gel containing 2.0 wt% of 4-armed-PEG-Mal and 2.0 wt% of the protein. Closed symbols: elasticity (G’) values and open symbols: viscosity (G”) values. Circles: TIP1 2C gel and triangles: TIP1 2C RGD gel. (TIF) [file pone.0107949.s005.tif]

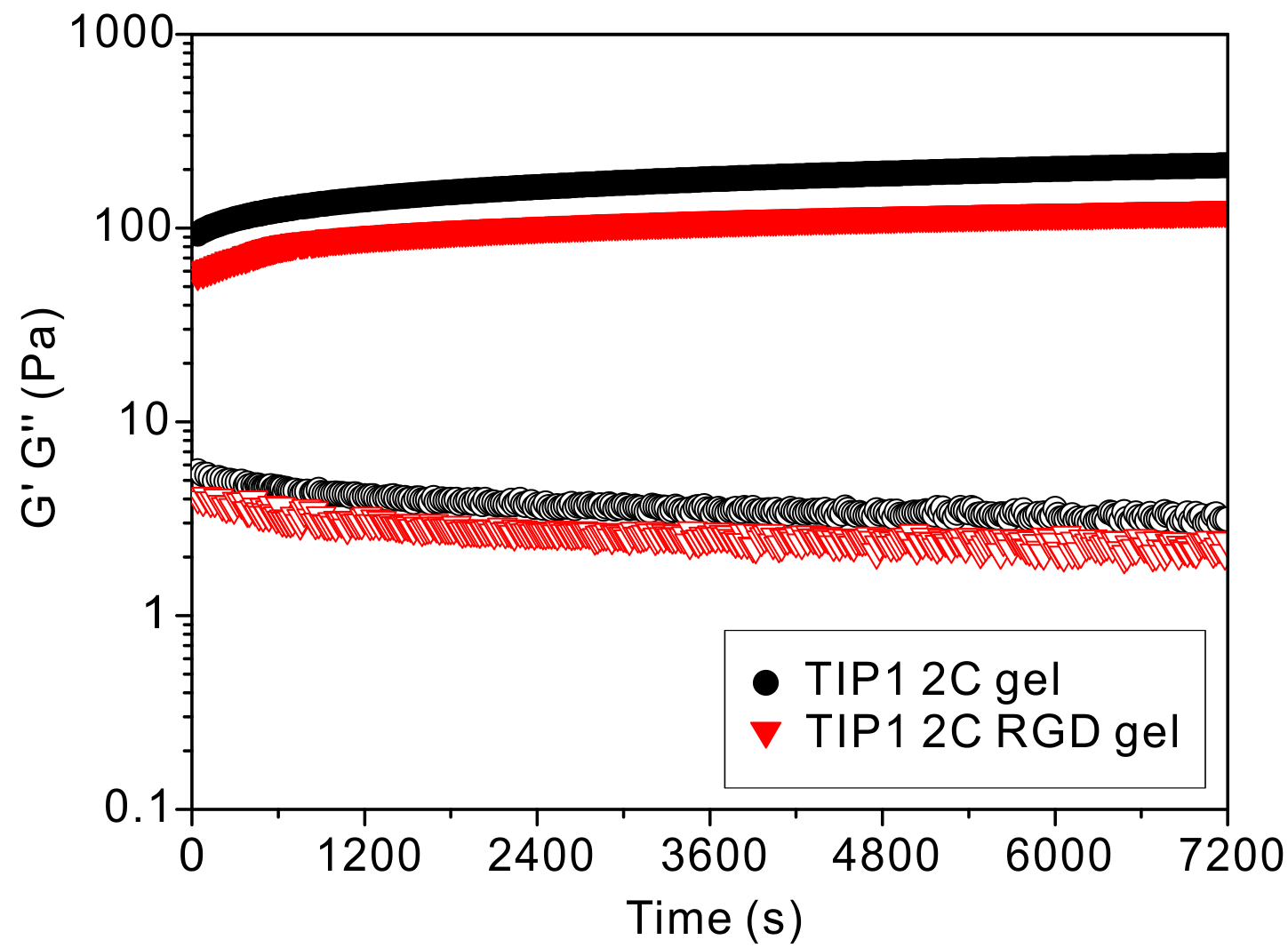

Supplement: Figure S6 — A dynamic time sweep of the gels. A rheological measurement in dynamic time sweep mode at the frequency of 1 rad/s and the strain of 1% for each gel containing 2.0 wt% of 4-armed-PEG-Mal and 2.0 wt% of the protein. Closed symbols: elasticity (G’) values and open symbols: viscosity (G”) values. Circles: TIP1 2C gel and triangles: TIP1 2C RGD gel. (TIF) [file pone.0107949.s006.tif]

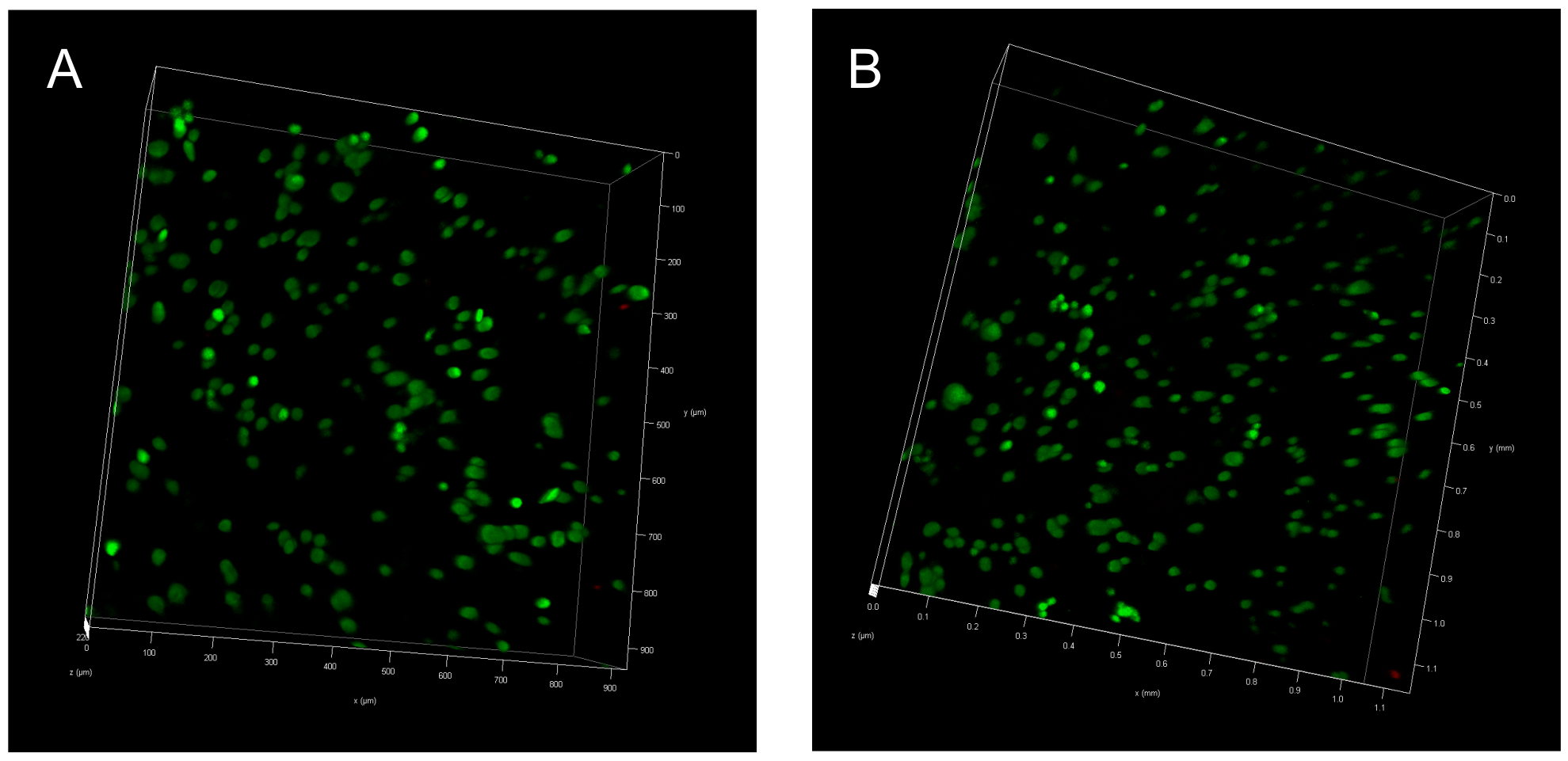

Supplement: Figure S7 — The cell distribution in both gels. Confocal images of 3D cell cultures in the hydrogels at day 1. Cells were distributed evenly in both gels. A, TIP1 2C gel and B, TIP1 2C RGD gel. (TIF) [file pone.0107949.s007.tif]

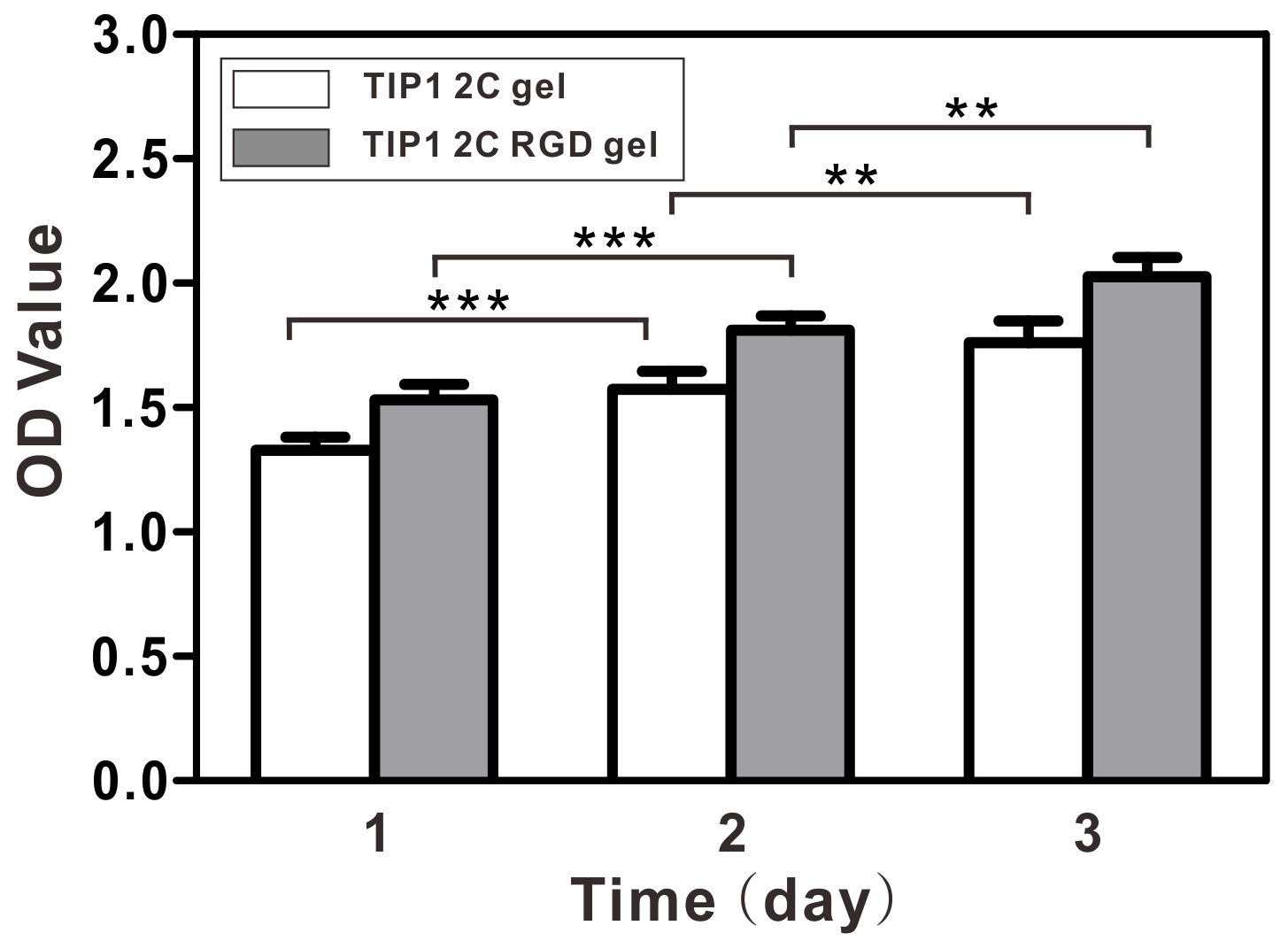

Supplement: Figure S8 — Determining cell proliferation. The cell proliferation rate of AD293 was evaluated by CCK-8 assay. Two asterisks (**) indicate a p value smaller than 0.01 (p<0.01). Three asterisks (***) indicate a p value smaller than 0.001 (p<0.001), n = 5. (TIF) [file pone.0107949.s008.tif]

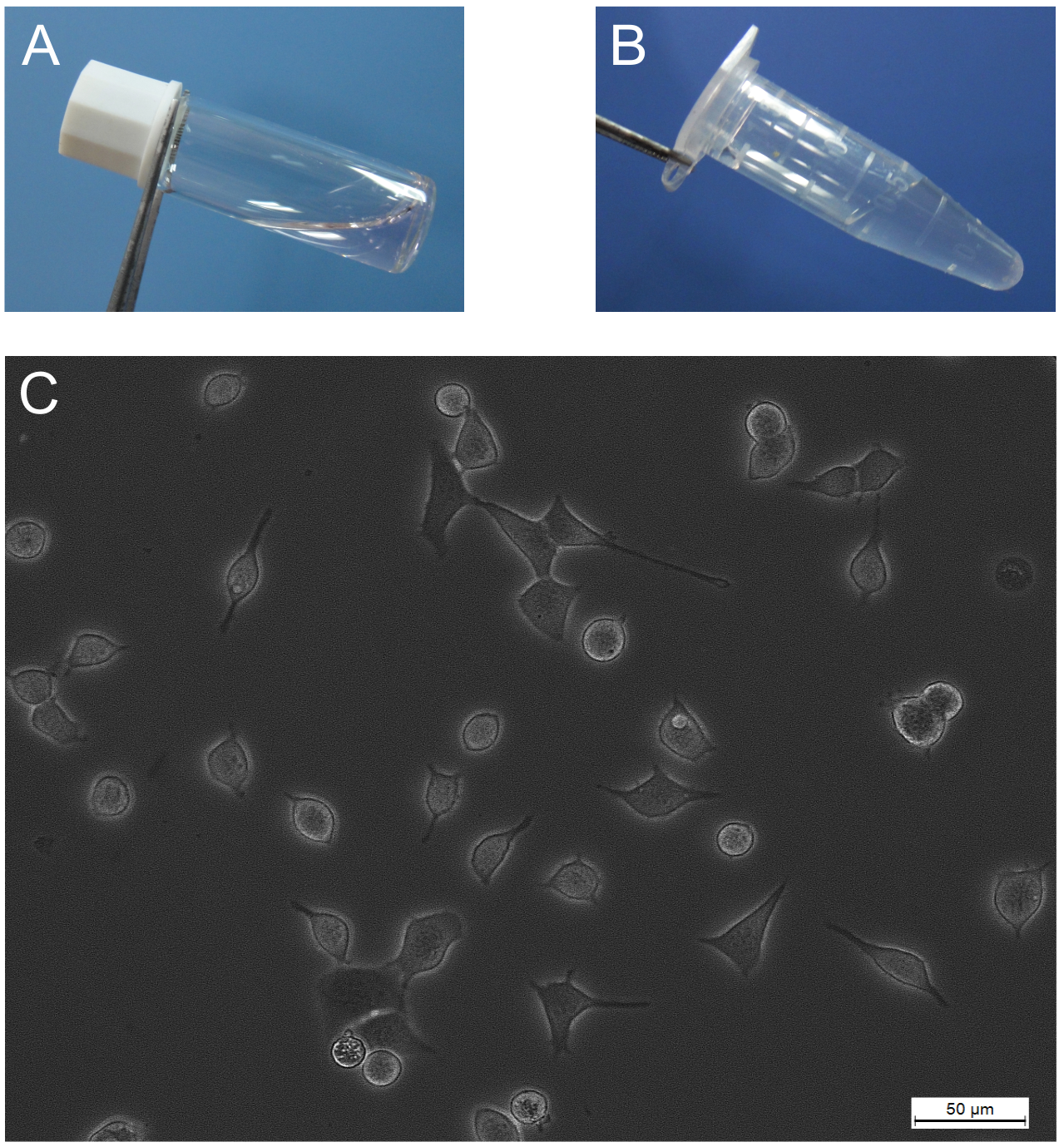

Supplement: Figure S9 — Cell Recovery. A, 200 µL of the TIP1 2C RGD gel was treated with 100 µL of trypsinase (2.5 µg/mL) for 5 minutes, and then 200 µL of PBS was added. B, The solution was then centrifuged at 1000 rpm for 5 minutes and no precipitation was observed. C, The AD293 cells were separated from the cell-gel construct by adding trypsinase and then centrifuging. The cells could grow well on a conventional 96-well culture plate. (TIF) [file pone.0107949.s009.tif]
