# Supplementary material for: A Genetically Modified Protein-Based Hydrogel for 3D Culture of AD293 Cells
Source: PLoS One. 2014 Sep 18;9(9):e107949. doi: 10.1371/journal.pone.0107949 (PMC4169439; doi:10.1371/journal.pone.0107949)
Supplement: Table S1 — The primers used for mutations in this study. The restriction sites are underlined. (DOC) [file pone.0107949.s010.doc]

Table S1. The primers used for mutations in this study. The restriction sites are underlined.

| primer name | primer sequence |
| --- | --- |
| TIP1 1 up  TIP1 T10C up  TIP1 S42C up  TIP1 S42C dn TIP1 S101C up  TIP1 S101C dn TIP1 S113C dn  TIP1 125 dn1  TIP1 125 dn2 | GCGAATTCATGTCCTACATCCCGGGCCAGCCGGTC  CCGGGCCAGCCGGTCTGCGCCGTGGTGCAAAG  GATCCTTGCCAGAATCCCTTCTC  GATTCTGGCAAGGATCCTGGTCG  CCAAGCGCTGCGAGGAGGTGGTGCG  CTCCTCGCAGCGCTTGGTGAGC  CACGGCCTTCTGCAGGCACTGCCGCGTCACCAG  GCCTCGAGCTAGGACAGCATGGACTGCTGCACGGCCTTCTGCAG  GCCTCGAGCTATGGGCTGTCTCCTCGTCCGGACAGCATGGACTGCTG |
